# The Differing Roles of Flavins and Quinones in Extracellular Electron Transfer in Lactiplantibacillus plantarum

**DOI:** 10.1128/aem.01313-22

**Published:** 2022-12-19

**Authors:** Joe G. Tolar, Siliang Li, Caroline M. Ajo-Franklin

**Affiliations:** a Department of BioSciences, Biomolecular Engineering Rice University, Houston, Texas, USA; b Department of Bioengineering, Biomolecular Engineering Rice University, Houston, Texas, USA; c Department of Chemical, Biomolecular Engineering Rice University, Houston, Texas, USA; Washington University in St. Louis

**Keywords:** extracellular electron transfer, flavins, lactic acid bacteria, metabolism, quinones

## Abstract

Lactiplantibacillus plantarum is a lactic acid bacterium that is commonly found in the human gut and fermented food products. Despite its overwhelmingly fermentative metabolism, this microbe can perform extracellular electron transfer (EET) when provided with an exogenous quinone, 1,4-dihydroxy-2-naphthoic acid (DHNA), and riboflavin. However, the separate roles of DHNA and riboflavin in EET in *L. plantarum* have remained unclear. Here, we seek to understand the role of quinones and flavins in EET by monitoring iron and anode reduction in the presence and absence of these small molecules. We found that addition of either DHNA or riboflavin can support robust iron reduction, indicating electron transfer to extracellular iron occurs through both flavin-dependent and DHNA-dependent routes. Using genetic mutants of *L. plantarum*, we found that flavin-dependent iron reduction requires Ndh2 and EetA, while DHNA-dependent iron reduction largely relies on Ndh2 and PplA. In contrast to iron reduction, DHNA-containing medium supported more robust anode reduction than riboflavin-containing medium, suggesting electron transfer to an anode proceeds most efficiently through the DHNA-dependent pathway. Furthermore, we found that flavin-dependent anode reduction requires EetA, Ndh2, and PplA, while DHNA-dependent anode reduction requires Ndh2 and PplA. Taken together, we identify multiple EET routes utilized by *L. plantarum* and show that the EET route depends on access to environmental biomolecules and on the electron acceptor. This work expands our molecular-level understanding of EET in Gram-positive microbes and provides additional opportunities to manipulate EET for biotechnology.

**IMPORTANCE** Lactic acid bacteria are named because of their nearly exclusive fermentative metabolism. Thus, the recent observation of EET activity—typically associated with anaerobic respiration—in this class of organisms has forced researchers to rethink the rules governing microbial metabolic strategies. Our identification of multiple routes for EET in *L. plantarum* that depend on two different redox active small molecules expands our understanding of how microbes metabolically adapt to different environments to gain an energetic edge and how these processes can be manipulated for biotechnological uses. Understanding the role of EET in lactic acid bacteria is of great importance due to the significance of lactic acid bacteria in agriculture, bioremediation, food production, and gut health. Furthermore, the maintenance of multiple EET routes speaks to the importance of this process to function under a variety of environmental conditions.

## INTRODUCTION

Microorganisms inhabit a rich diversity of environmental niches, including those with highly limited resources. Consequently, some bacteria have developed unique metabolic strategies to survive in resource-poor environments. One such strategy is extracellular electron transfer (EET), which allows cells to achieve redox balance by transferring electrons out of the cell to terminal electron acceptors in their environment via an electron transfer network (1, 2). Historically, this ability was thought to be limited to Gram-negative microbes because of the presence of a large, insulated cell wall in Gram-positive microbes (3). However, it was recently discovered that Listeria monocytogenes, a Gram-positive opportunistic human pathogen, utilizes a flavin-based EET pathway (4–6) that is linked to a genetic locus (the FLEET locus). This allowed us and others to identify other Gram-positive microbes capable of EET, including Enterococcus faecalis and Lactiplantibacillus plantarum (5, 7–9). At present, however, it is not understood how primarily fermentative microbes, such as *L. plantarum* and E. faecalis, can utilize and maintain an EET pathway, especially when a traditional respiratory system is not maintained. Elucidation of this phenomenon and its influence on organismal physiology is of great importance due to the prevalence of these Gram-positive bacteria in agriculture, bioremediation, food production, gut health, and opportunistic human infections (10–13).

Prior work in other bacteria provides insight into the possible roles of different redox active small molecules, such as quinones and flavins, in EET. In L. monocytogenes, electrons are transferred from intracellular NADH to a membrane-confined quinone pool via Ndh2, a type II NADH dehydrogenase, then to an extracellular membrane-anchored flavolipoprotein, PplA, and finally to terminal electron acceptors, including ferric iron and electrodes (5). A number of studies have also found quinone derivatives can support EET by functioning as extracellular electron shuttles between microbes and insoluble electron acceptors (14–16). Additionally, flavins are known to mediate or support extracellular electron transfer in *Clostridium*, *Shewanella*, *Geobacter*, and other microbes (17–22).

We recently showed that the genus *Lactiplantibacillus* has a highly conserved FLEET locus, and many of its members exhibit EET activity (9). The FLEET locus encodes a type II NADH dehydrogenase (Ndh2), flavin transport proteins (FmnA/B and ATPase 1/2), membrane demethylmenaquinone (DMK) synthesis proteins (DmkA and EetB/DmkB), and an electron transfer pathway that contains a flavin mononucleotide (FMN) cofactor (PplA) and EetA (7–9). An exogenous menaquinone (MK) precursor, 1,4-dihydroxy-2-naphthoate (DHNA), was added to allow the microbe to synthesize MK or DMK. When both an electron acceptor (ferric iron or an extracellular electrode) and DHNA were provided, EET activity was observed in *L. plantarum*. Additionally, increasing the concentration of riboflavin increased EET activity (9). Closer analysis of the FLEET locus across *L. plantarum* strains found that transposon insertions in either *ndh2* or *pplA* caused a loss of iron reduction (9). While establishing the importance of riboflavin and DHNA in EET in *L. plantarum*, the precise role of these molecules in extracellular electron transfer remains unclear.

Through a series of electrochemical assays coupled with genetic knockouts, this study identifies and characterizes two distinct pathways for EET in *L. plantarum*. We found that both riboflavin and DHNA can independently support robust EET but differ in the extent to which they support iron reduction and anode reduction. Here, we show functional Ndh2, PplA, and EetA are required for flavin-dependent EET, while only Ndh2 and PplA are necessary for DHNA-dependent EET. Additionally, we found that DHNA can act as a robust electron shuttle between microbes and a carbon felt electrode, while riboflavin is less efficient as an electron shuttle under the same conditions. Overall, we have identified distinct electron transfer mechanisms that utilize commonly found redox-active small molecules and that may have evolved to provide interchangeable energetic means to adapt to different environments.

## RESULTS

### *L. plantarum* uses either DHNA or riboflavin to support extracellular electron transfer.

Prior work by Tejedor-Sanz et al. (9) has demonstrated *L. plantarum* requires PplA to reduce iron but not for electrode reduction. Specifically, PplA was required for iron reduction when cells were assayed in phosphate-buffered saline (PBS), a condition that does not permit cell growth. In contrast, PplA was not required for anode reduction when cells were suspended in chemically defined media, a condition that enables cell growth. Thus, the differential requirement of PplA could be caused by the difference in the electron acceptor or because cells were prepared and grown differently, depending on the assay used (9). Recently, studies of extracellular electron transfer in other organisms that contain the FLEET locus, L. monocytogenes (5) and E. faecalis (8, 23), suggested such requirements may be dependent on growth conditions. Thus, we sought to understand how growth conditions affect whether DHNA and riboflavin are necessary to support iron reduction.

**FIG 1:**
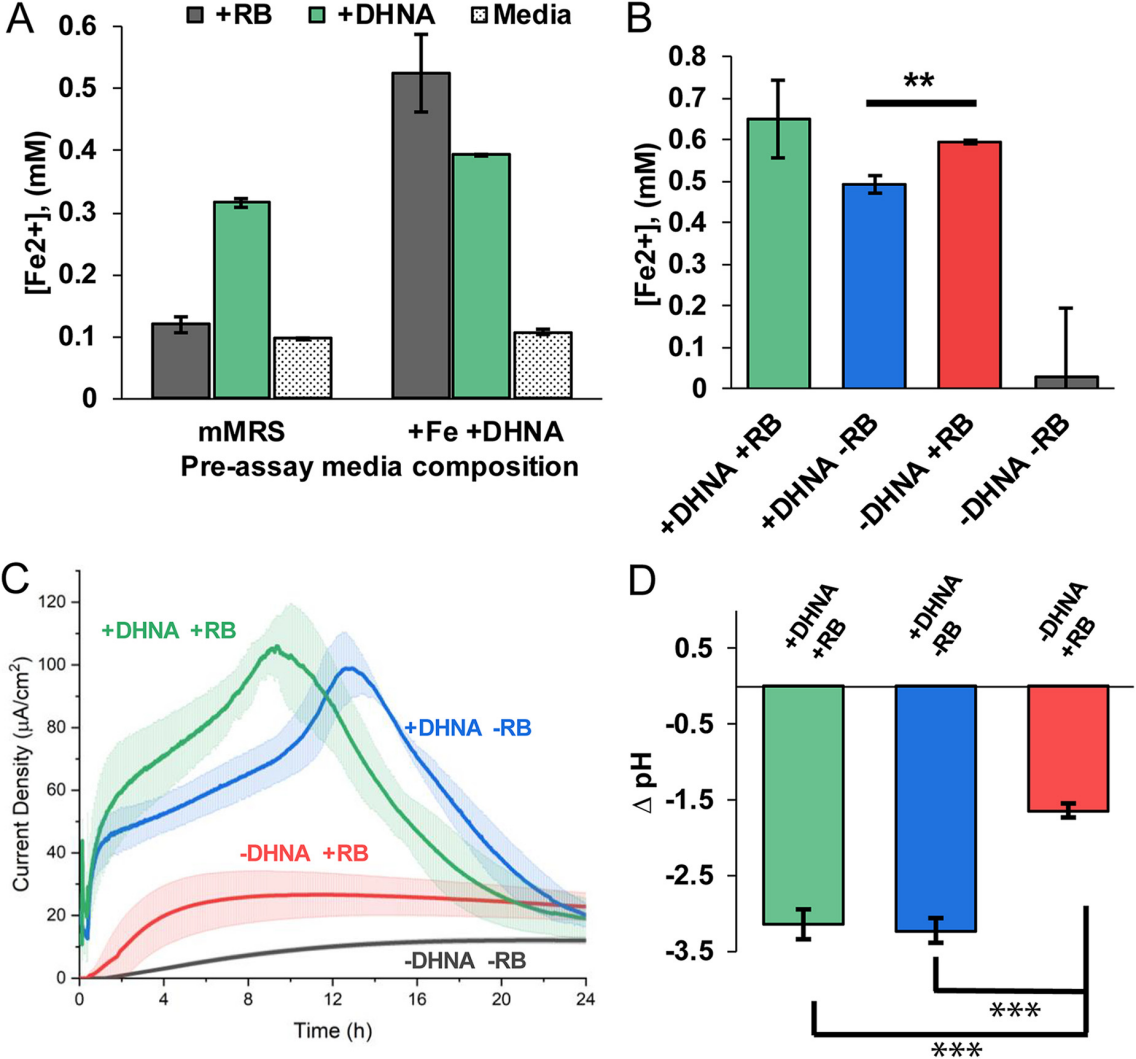
DHNA or flavins can support EET activity in distinct ways. (A) Concentration of Fe^2+^ produced from ferric oxide nanoparticles after 24 h of anaerobic incubation in PBS plus 20 mg/mL mannitol (stippled) alone or supplemented with DHNA (20 μg/mL [green]) or riboflavin (RB) (2 μg/mL [gray]). The preassay medium composition indicates the overnight culture source contained exogenous DHNA and ferric ammonium citrate or was unsupplemented. (B) Concentration of Fe^2+^ produced from ferric oxide after 24 h of anaerobic incubation in PBS plus 20 μg/mL mannitol with DHNA and riboflavin (green) compared to DHNA (blue) or riboflavin (red) alone. (C) *L. plantarum* was grown in microaerobic 3-chamber bioelectrochemical reactors for 24 h on an electrode poised at 0.2 V versus Ag/AgCl (3 M KCl). Chronoamperometric measurements were taken every 36 s. Error bars show standard deviation (SD). (D) After 24 h, the pH of the medium was measured and is reported as the change in pH compared to the starting pH of 7.4. *n*= 3 for all experiments. Statistical significance was calculated with a *t* test (***, *P* < 0.001).

To test this, we first grew cells in mMRS alone (see Materials and Methods) or in mMRS supplemented with ferric ammonium citrate and DHNA. Cells from each preassay condition were washed, incubated in media with 20 μg/mL DHNA or 2 μg/mL riboflavin, and then assayed for their ability to reduce iron(III) oxide nanoparticles under anaerobic conditions (see Fig. S1 in the supplemental material). We found that preassay medium composition had no effect on iron reduction in wells lacking DHNA (−DHNA) or riboflavin (−RB) as well as those containing DHNA (+DHNA) (Fig. 1A). Conversely, we found that preassay medium supplemented with DHNA plus iron supported robust iron reduction activity under riboflavin-containing assay conditions (Fig. 1A). We also found that supplementing the preassay medium with only DHNA was sufficient to enable the riboflavin-dependent iron reduction (Fig. S2). These results show that when grown with ferric ammonium citrate and DHNA, *L. plantarum* can use either riboflavin or DHNA to reduce iron. Since supplementing preassay medium with both ferric ammonium citrate and DHNA resulted in the most robust iron reduction, all of the following experiments were conducted under those conditions.

Because DHNA and riboflavin both have midpoint potentials more negative than that of iron(III) oxide, it is possible that their effects on iron reduction could be additive. To determine whether iron reduction by riboflavin and DHNA could be additive, we measured iron reduction by *L. plantarum* in medium with DHNA and riboflavin(+DHNA +RB), DHNA alone (+DHNA), riboflavin alone (+RB), and medium lacking both (−DHNA −RB). As expected, *L. plantarum* lacking both riboflavin and DHNA did not reduce iron (Fig. 1B). In contrast, combining riboflavin and DHNA did not have an additive effect on iron reduction (Fig. 1B). Together, these results indicate that, under these experimental conditions, iron reduction by *L. plantarum* proceeds at approximately equal rates through a DHNA- or flavin-dependent pathway.

While iron reduction provides researchers with a method for large-scale screening, EET activity can be measured with exquisite temporal resolution via the reduction of a poised electrode. Many studies have shown that EET activity can vary between iron reduction and anode reduction (9, 23). Because extracellular iron reduction is supported by riboflavin in the absence of DHNA, we decided to test if riboflavin can support anode reduction when reactor conditions mimic iron reduction conditions. To do this, we replaced iron(III) oxide with an anode biased at +0.20 V versus Ag/AgCl and probed current production by DHNA and riboflavin alone or in combination, alongside heat-killed *L. plantarum*, or alongside viable cells. Chronoamperometry of only DHNA, only riboflavin, or DHNA and riboflavin showed that abiotic oxidation of DHNA occurs, but it decays exponentially (Fig. S3). Riboflavin did not reduce the electrode (Fig. S3), as expected since it is introduced in its oxidized form. Likewise, when heat-killed cells were introduced into buffer containing DHNA or riboflavin, the background current was only 1 to 2 μA/cm^2^ (Fig. S3). These data show that minimal current is produced without metabolically active cells.

Living *L. plantarum* cells without DHNA or riboflavin steadily produce ~12 μA/cm^2^ of current (Fig. 1C), indicating a low level of EET occurs without these exogenous molecules. In contrast, when cells were added to DHNA-containing medium, the current rapidly increased to reach a maximum current density of ~100 μA/cm^2^ within 10 to 13 h and then gradually decreased. In riboflavin-supplemented medium, the current gradually increased from viable cells and reached a sustained plateau by ~4 h. With riboflavin, the maximum current density was ~4-fold lower than that in medium containing DHNA, but it was still significantly higher than that in cells without either DHNA or riboflavin (Fig. 1C). These data indicate that riboflavin and DHNA can both support significant EET to iron or an anode. However, the differing rates and time evolutions of flavin-dependent and DHNA-dependent EET to a poised electrode suggest this EET may occur by different mechanisms.

While it is clear that DHNA supports more robust current production than riboflavin, the physiological implications remain unclear. EET activity in *L. plantarum* was recently found to accelerate fermentative metabolism and resulted in a drop of extracellular pH (9). To see if the DHNA- and riboflavin-dependent routes equally contribute to the fermentative flux, we measured the pH of the medium after 24 h and found that the change in pH was significantly smaller in the riboflavin-containing medium than in DHNA-containing medium (Fig. 1D). These data suggest that DHNA-dependent EET is more closely linked to the fermentative flux observed by Tejedor-Sanz et al. (9) than flavin-dependent EET.

### DHNA acts as a robust, reversible electron shuttle.

**FIG 2:**
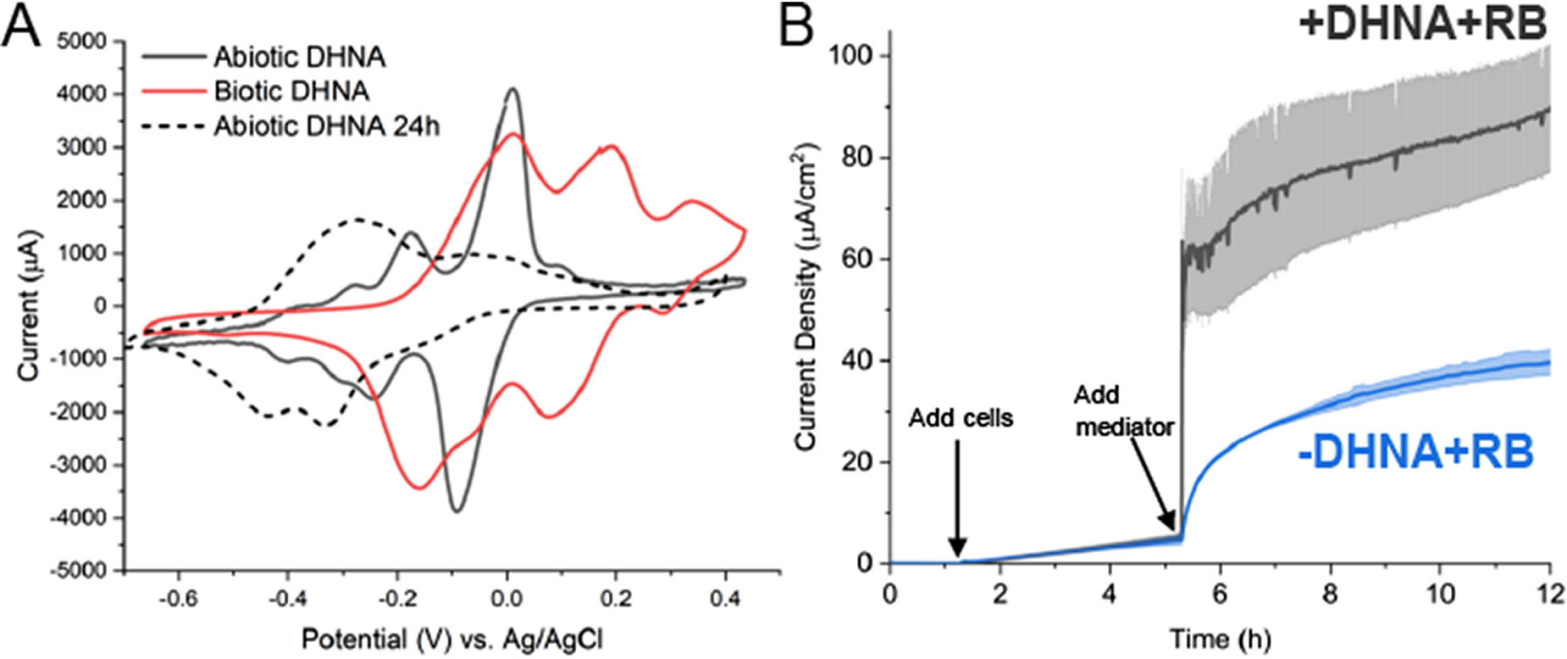
DHNA exhibits redox shuttle characteristics. (A) Cyclic voltammetry was performed prior to the addition of cells and 24 h after the addition of cells. (B) *L. plantarum* was grown in microaerobic 3-chamber bioelectrochemical reactors for 3 h on an electrode poised at 0.2 V versus Ag/AgCl (3 M KCl) before riboflavin (blue) or DHNA and riboflavin (gray) was injected. Current density measurements were taken every 36 s for 12 h. *n* = 3 bioreactors, and error bars show standard deviation.

Having established that DHNA supports both anode and iron reduction by *L. plantarum*, we next sought to characterize the mechanism of DHNA-dependent anode reduction. Our first step was to distinguish between two ways DHNA could support EET: either by acting intracellularly as a precursor for DMK, which is a membrane-confined electron carrier in EET in L. monocytogenes (5) or, DHNA could be acting as a mediator, much like its close analog 2-amino-3-carboxy-1,4-naphthoquinone (ACNQ) (24). To evaluate the two hypotheses, we performed cyclic voltammetry in reactors containing freshly dissolved DHNA, DHNA that had been in the reactor for 24 h, and DHNA incubated with cells for 24 h. We found that freshly dissolved DHNA, which imparts a green tint to the solution, has two distinct redox peaks (−175 mV and −11 mV versus Ag/AgCl) that correspond to literature values (Fig. 2A). Interestingly, under abiotic conditions over 24 h, the DHNA solution became slightly pink and exhibited a redox shift to approximately −320 mV (versus Ag/AgCl), which is in line with its conversion to ACNQ (Fig. 2A) (24). Most profoundly, we found that DHNA in the presence of bacteria remains green in tint and exhibits the −11 mV peak (versus Ag/AgCl) and an additional peak at approximately +196 mV (versus Ag/AgCl) (Fig. 2A). These data suggest that DHNA acts as a mediator and is predominantly reduced in the presence of bacteria.

To further probe this possibility, we removed the cells from the media with and without DHNA after 24 h and again performed cyclic voltammetry on the spent media (Fig. S4). The spent medium from cells without DHNA did not have any significant peaks in the cyclic voltammogram. In contrast, spent medium from cells with DHNA showed the same two peaks (at −11 mV and +196 mV) as the cells with DHNA after 24 h (Fig. 2A). The stretching of the redox peak for DHNA in the presence of cells suggests DHNA is being rereduced, likely by the cells. These data strongly suggest that DHNA can serve as a reversible redox mediator for *L. plantarum* under these conditions.

We reasoned that DHNA acting as a mediator will enable EET more rapidly than DHNA acting as a precursor for DMK. Thus, we next performed a pulse-chase experiment to observe the real-time bioelectrochemical response to the addition of DHNA. After a 3- to 4-h acclimation period for injected cells, either riboflavin or DHNA plus riboflavin was added and the current production was sampled for 12 h. The addition of DHNA plus riboflavin resulted in a large, immediate jump in current production, whereas the addition of just riboflavin resulted in a much slower increase in current production (Fig. 2B). To quantify the time required to utilize DHNA, we calculated the amount of time required to reach half the maximal current, *t*_1/2max_, for two concentrations of DHNA and found that DHNA induced responses with half-times of ~14 s at 20 μg/mL and ~800 s at 0.2 μg/mL (Table 1). This time scale is most consistent with a freely diffusible molecule (25), providing additional support for DHNA’s role as a mediator.

**TABLE 1:** Quantitative characterization of EET reaction rate to DHNA and riboflavin^*a*^

Condition	Mean ± SD max current density (μa/cm^2^)	Half-time to max current density (s)
DHNA		
0.2 μg/mL	15.86 ± 2.82	792
20 μg/mL	70.36 ± 1.55	14.4
Riboflavin		
0.2 μg/mL	24.32 ± 0.22	3,096
2 μg/mL	45.02 ± 1.80	2,988
20 μg/mL	55.17 ± 5.26	684

a *L. plantarum* was grown in microaerobic 3-chamber bioelectrochemical reactors for 3 h on an electrode poised at 0.2 V versus Ag/AgCl (3 M KCl) before the indicated concentration of riboflavin or DHNA was injected. Current density measurements were taken every 36 s for 24 h. Half-time was determined as 1/2 the time at which maximum current was achieved. Data are representative of 3 bioreactors.

As a last confirmation, we performed a medium swap experiment: DHNA was supplied to cells in a bioelectrochemical reactor, and after ~16 h, the cells were collected, washed, and inoculated into a second bioelectrochemical reactor with fresh medium lacking DHNA. Because *L. plantarum* cannot be removed from a carbon felt electrode, we used graphite rods as the working electrode in these bioelectrochemical reactors. As before, DHNA enabled significant current production. (The magnitude of this current density was lower than what is observed with carbon felt, which arises from the difference in microbially accessible surface area between the two types of electrodes.) Swapping the media to remove DHNA abolished the generation of current; the addition of fresh DHNA restored current production (Fig. S5). Taken together, these data indicate DHNA can act as a freely diffusing redox mediator that receives electrons and can donate them to a poised electrode.

### DHNA-dependent EET requires Ndh2 and PplA.

**FIG 3:**
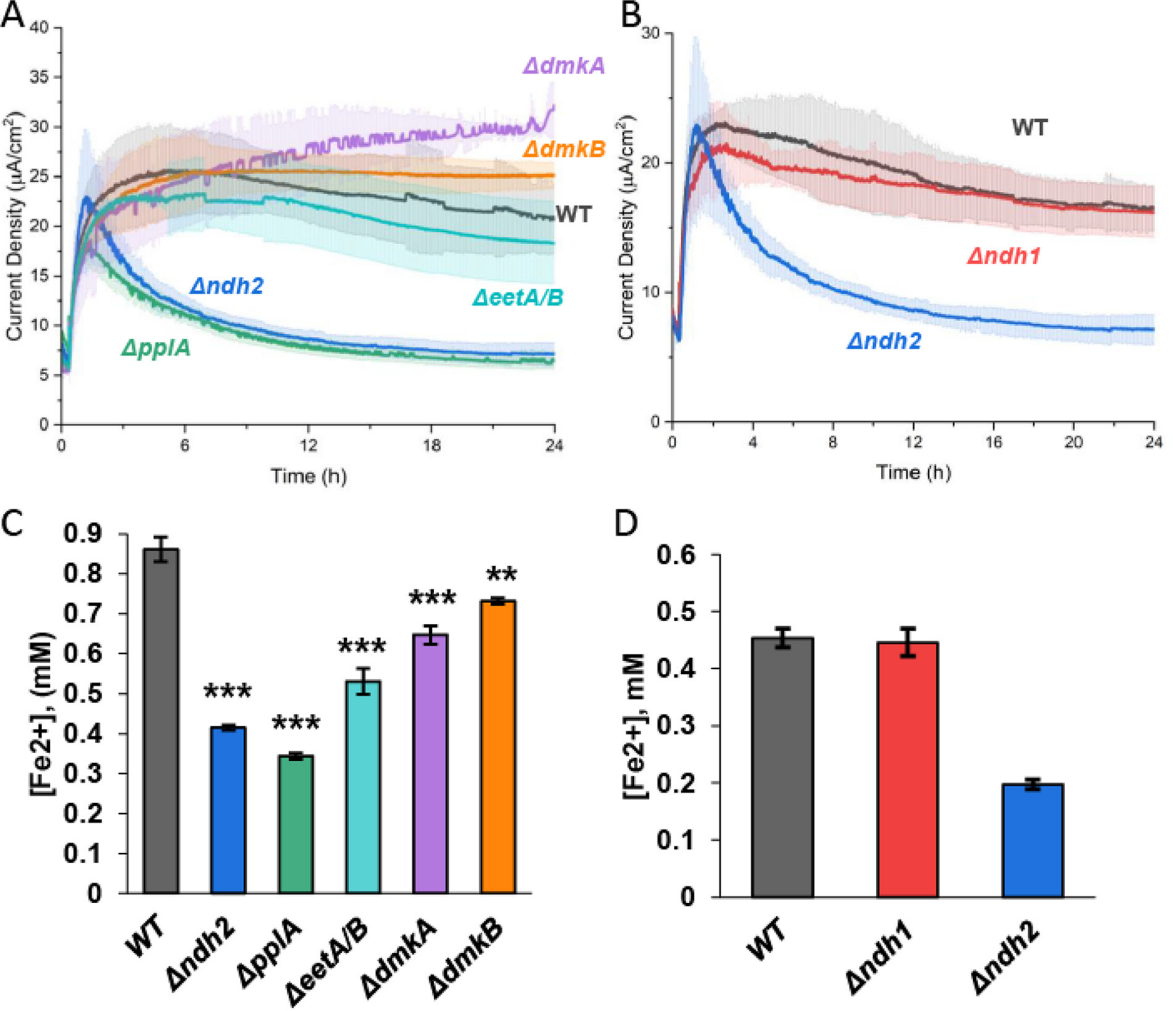
DHNA-dependent EET utilizes Ndh2 and PplA to reduce extracellular electron acceptors. Chronoamperometry was performed in 2-chamber 3-electrode bioreactors. (A) WT level current production in DHNA (20 μg/mL)-containing medium was supported under all conditions (Δ*eetA*/*B*, Δ*dmkA*, and Δ*dmkB* mutants) except for the Δ*ndh2* and Δ*pplA* mutants. (B) The Δ*ndh1* mutation was not required for DHNA-dependent current production. (C) Iron oxide nanoparticle reduction was assayed after 24 h of anaerobic incubation in DHNA-containing media with WT, Δ*ndh2*, Δ*pplA*, Δ*eetA*/*B*, Δ*dmkA*, and Δ*dmkB* strains. We found that the Δ*ndh2* and Δ*pplA* mutants have the largest decrease in reduced iron, followed by the Δ*eetA*/*B* mutant and then the Δ*dmkA* and Δ*dmkB* strains. (D) Δ*ndh1* mutation was not required for DHNA-dependent iron reduction. *n* = 3 for all experiments. All errors shown are SD. Statistical significance was calculated by *t* test (*, *P* < 0.05; ***, *P* < 0.001).

With the identification of a DHNA-dependent route for anode reduction that appears to utilize DHNA as an extracellular electron shuttle, we wanted to characterize which FLEET genes were required to support anode reduction. When cells are growing, Ndh2 and PplA are necessary for iron reduction, but only Ndh2 is required for anode reduction (9). To identify which FLEET genes were required to perform DHNA-dependent EET when cells are resting rather than growing, knockouts of Ndh2, PplA, EetA/B, DmkA, and DmkB were created using the CRISPR-Cas9/RecET system (26). We then compared the DHNA-dependent current produced by these mutants to that produced by wild-type (WT) cells (Fig. 3A and B) and what was expected for DHNA-independent or abiotic EET. To minimize pH changes over the course of the experiment, we halved the cell density in these experiments (optical density at 600 nm [OD_600_] = 0.25) relative to the experiments shown in Fig. 1 (OD_600_ = 0.5).

In the presence of 20 μg/mL DHNA, WT cells produced ~25 μA/cm^2^ (Fig. 3A), which is in line with what we expect for half the number of cells used in the prior experiment (+DHNA −RB trend in Fig. 1C). On the basis of prior data, we expect DHNA-independent EET to result in ~6 μA/cm^2^ of current (i.e., one-half the current of the −DHNA −RB condition shown in Fig. 1C). Additionally, DHNA produces abiotic current of ~1.5 μA/cm^2^; therefore, we expect the total abiotic and DHNA-independent baseline current in this experiment to approach ~8 μA/cm^2^. When the mutants were tested for their ability to produce current, the Δ*dmkA*, Δ*dmkB*, and Δ*eetA*/*B* mutants showed similar current output compared to WT (Fig. 3A). Conversely, the Δ*ndh2* and Δ*pplA* mutants had an ~2-fold decrease in current compared to the WT (Fig. 3A), which was comparable to the expected baseline current. These data confirm the importance of Ndh2 under multiple experimental conditions. Additionally, these data show PplA can be required for anode reduction under nongrowth conditions, whereas it was not required under conditions that promoted cell growth. Given the importance of *ndh2*, we also deleted *ndh1*, a second, type-II NADH dehydrogenase in *L. plantarum.* This deletion had no effect on current production compared to WT (Fig. 3B), indicating *ndh1* is not involved in EET. Thus, the DHNA-dependent anode reduction proceeds primarily through Ndh2 and PplA with DHNA acting as a redox mediator (see Fig. 6 below).

Having established the role of FLEET genes in DHNA-dependent anode reduction, we next sought to address the involvement of these genes in DHNA-dependent iron reduction. To understand which FLEET genes are required to reduce iron, we tested each mutant's ability to reduce iron oxide nanoparticles under anaerobic conditions. Consistent with previous work, both of the Δ*ndh2* and Δ*pplA* mutants showed a significant decrease in iron reduction compared to WT cells (Fig. 3C). We also found that the Δ*dmkA* and Δ*dmkB* mutants exhibited a minor but significant decrease in reduced iron, while Δ*eetA*/*B* cells were found to have a slightly larger decrease in reduced iron than the WT (Fig. 3C). As seen in anode reduction, Δ*ndh1* cells were found to have no significant change in iron reduction compared to WT cells in the presence of DHNA (Fig. 3D).

Since the deletion of *ndh2*, *pplA*, *eetA*, and *eetB* could impair iron reduction via polar effects, we sought to complement their expression and examine iron reduction of the complemented mutant strains. We were able to generate strains that expressed *ndh2*, *eetA*, and *eetB* in *trans*. We found that in a Δ*dmkA* Δ*ndh1* Δ*ndh2* strain, expression of Ndh2 resulted in iron reduction comparable to that of WT cells (Fig. S6A). Additionally, we found that in a Δ*eetA*/*B* strain, EetA complementation resulted in a small significant increase in iron reduction compared to the Δ*eetA*/*B* strain, but neither EetA nor EetB could restore WT level DHNA-dependent iron reduction (Fig. S6B). Together, these data indicate that extracellular electron transfer begins at Ndh2 via the oxidation of cytosolic NADH. As a working model, we suggest that electrons can then be directly transferred to DHNA that is then shuttled extracellularly to PplA via EetA; ultimately reducing ferric iron or an anode (Table 2 and see Fig. 6 below).

**TABLE 2:** Genetic requirements of EET activity

Gene	Requirement for EET activity^*a*^:			
	DHNA dependent		Flavin dependent	
	Iron reduction	Anode reduction	Iron reduction	Anode reduction
*ndh2*	+	+	+	+
*eetA* or *eetB*	+	−	+	+
*pplA*	+	+	−	+
*ndh1*	−	−	−	−
*dmkA*	−	−	−	−
*dmkB*	−	−	−	−

a +, required; −, not required.

### Riboflavin mediates electron transfer through an unknown, protein-mediated mechanism.

**FIG 4:**
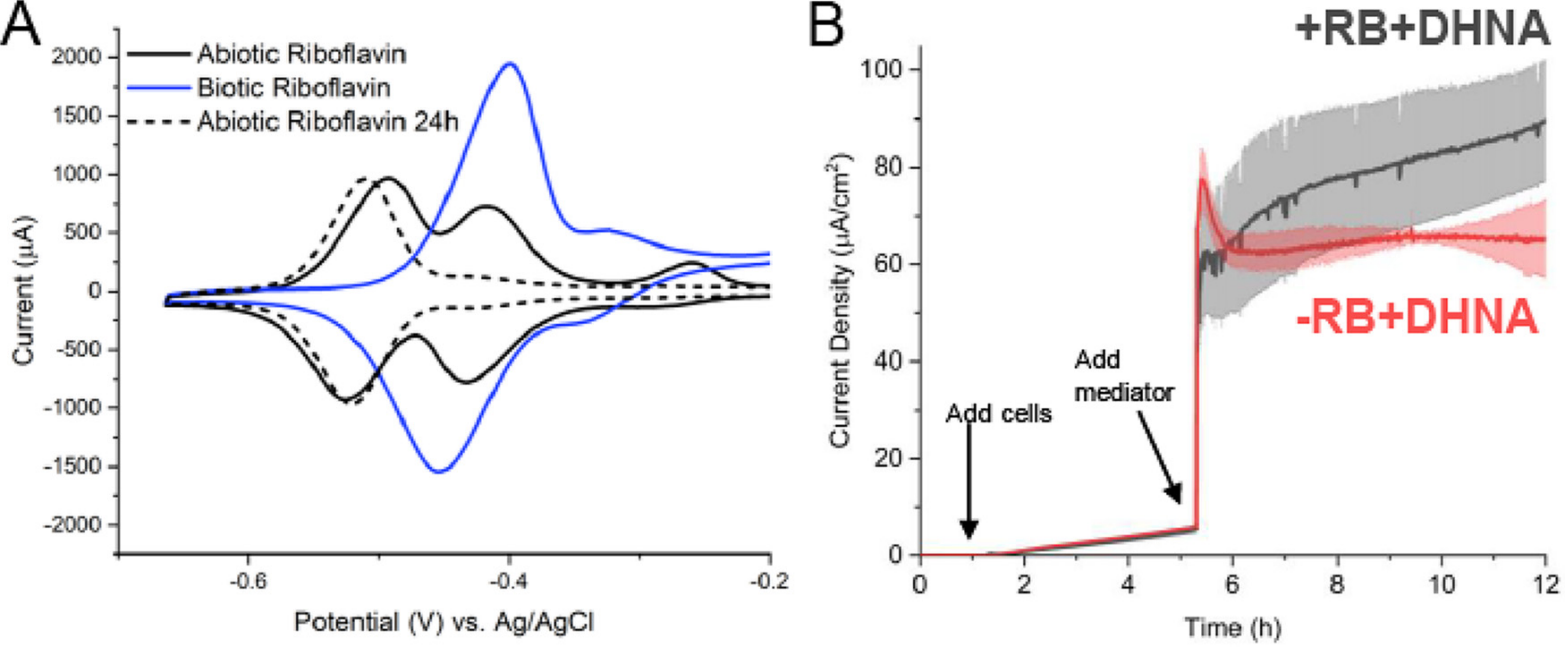
Riboflavin exhibits poor redox shuttle characteristics. (A) Cyclic voltammetry was performed prior to the addition of cells and 24 h after the addition of cells. A positive potential shift is observed for riboflavin in the presence of cells. (B) *L. plantarum* was grown in microaerobic 3-chamber bioelectrochemical reactors for 3 h on an electrode poised at 0.2 V versus Ag/AgCl (3 M KCl) before DHNA (red) or DHNA plus riboflavin (gray) was injected. Current density measurements were taken every 36 s for 12 h. *n* = 3 reactors, and error bars show SD.

Having established the DHNA-dependent EET pathway in *L. plantarum*, we turned to interrogation of the flavin-dependent EET pathway. The differential responses to DHNA and riboflavin we observed suggest that DHNA could be acting as an electron shuttle between the microbe and the electrode, while riboflavin likely requires a longer exposure to elicit its effect. To determine if riboflavin is acting as a mediator during flavin-dependent EET, we performed cyclic voltammetry on media containing freshly dissolved riboflavin, riboflavin that had been in the reactor for 24 h, and riboflavin incubated with cells for 24 h. Freshly dissolved riboflavin has two prominent redox peaks at −493 mV and −416 mV (versus Ag/AgCl) (Fig. 4A). Interestingly, 24 h on a poised electrode under microaerobic conditions resulted in the loss of the peak at −416 mV (versus Ag/AgCl), while the peak at −510 mV (versus Ag/AgCl) persisted (Fig. 4A). Conversely, the presence of cells resulted in the loss of the peak at −493 mV (versus Ag/AgCl) and increased the amplitude of the redox peak at −399 mV (versus Ag/AgCl) (Fig. 4A). This biotic shift suggests that cells may be converting riboflavin to FMN, similar to results seen in previous studies (20).

Conversion of riboflavin to FMN to be used in subsequent processes by *L. plantarum* would require more time than immediate utilization of riboflavin as a mediator. To interrogate the time scale of response to the addition of riboflavin, we performed chronoamperometry following the addition of riboflavin plus DHNA and DHNA alone. We found that the addition of riboflavin plus DHNA did not result in an immediate increase in current compared to DHNA alone, but it did enable a greater maximum current density that was reached at ~16 h (Fig. 4B). This finding strongly suggests that riboflavin does not act immediately to support anode reduction, but rather it enhances EET by an unknown mechanism that requires time to incorporate, transport, or modify riboflavin.

We next sought to characterize the degree to which concentration can affect riboflavin’s ability to support EET using pulse-chase chronoamperometry. We found that the maximum current density increased with the riboflavin concentration, and there was a proportional decrease in the time to ½ the maximal current (Table 1). Overall, riboflavin-supported current production was less robust than DHNA-dependent EET. Interestingly, the increase in riboflavin concentration did not result in a proportional increase in current density, suggesting that cells may only utilize a particular concentration. In summary, it is likely that riboflavin could be supporting EET, in part, as an electron shuttle, or as some form of cofactor.

To probe if riboflavin can serve as an electron shuttle, we performed another medium swap using a graphite rod electrode, similar to the approach used with DHNA, to select for mediated electron transfer. We found that riboflavin did support current production and that the current was dependent on the presence of riboflavin (Fig. S7). These data suggest that riboflavin can serve as a shuttle under conditions that promote mediated electron transfer, but the primary mechanism by which riboflavin supports EET is likely by enhancing direct electron transfer rather than by acting as a mediator (see Fig. 6 below).

### Flavin-mediated EET requires Ndh2, PplA, and EetA.

**FIG 5:**
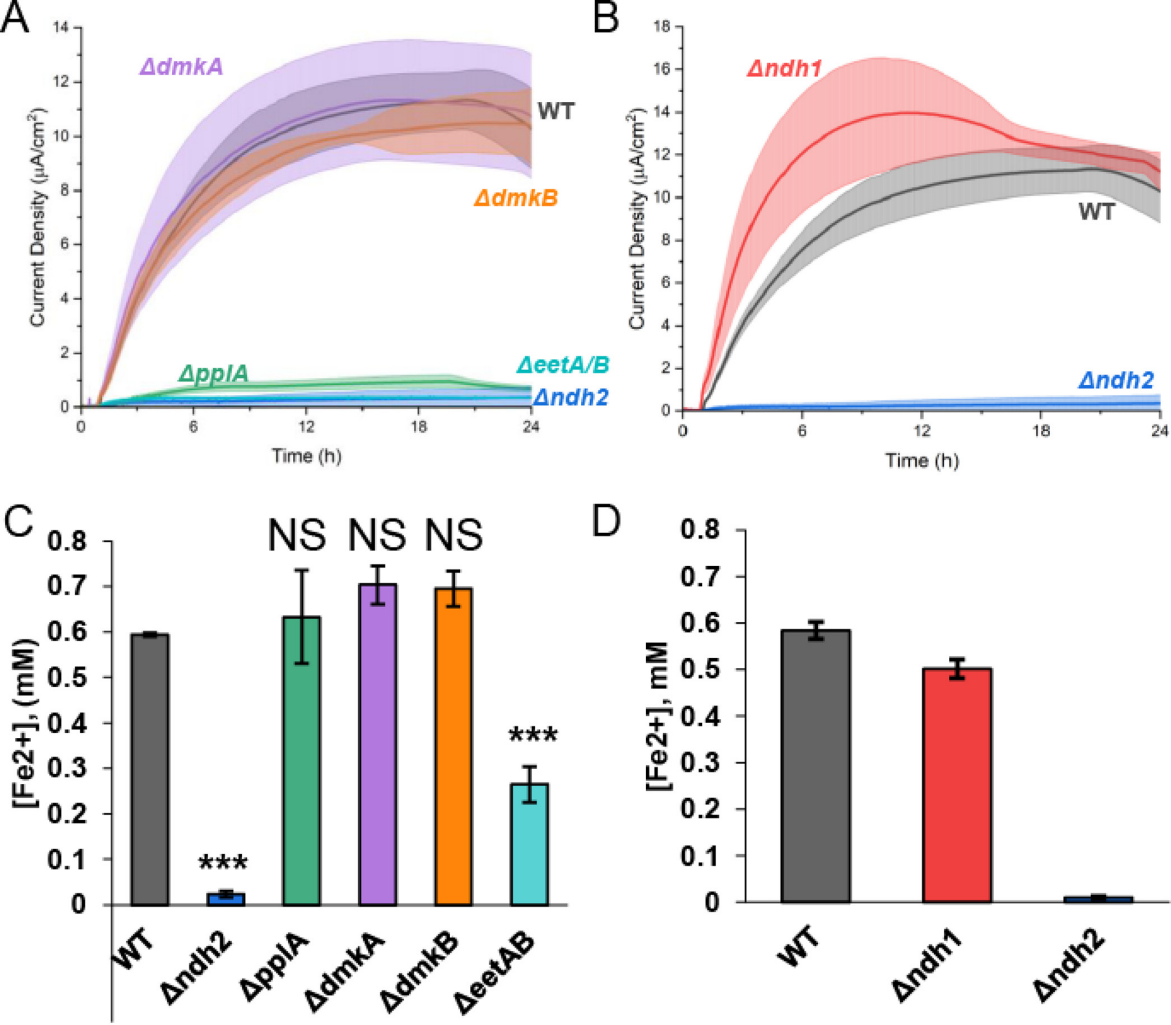
Flavin-dependent EET utilizes Ndh2, EetA, and PplA to reduce extracellular electron acceptors. Chronoamperometry was performed in 2-chamber 3-electrode bioreactors. (A) WT-level current production in riboflavin (2 μg/mL)-containing medium was supported by the Δ*dmkA* and Δ*dmkB* mutants, while the Δ*ndh2*, Δ*eetA*/*B*, and Δ*pplA* mutants were unable to produce current. (B) Δ*ndh1* mutation was not required for riboflavin-dependent current production and resulted in improved initial current production. (C) Iron oxide nanoparticle reduction was assayed after 24 h of anaerobic incubation in DHNA-containing medium with the WT and Δ*ndh2*, Δ*pplA*, Δ*eetA*/*B*, Δ*dmkA*, and Δ*dmkB* mutants. We found that the Δ*pplA*, Δ*dmkA*, and Δ*dmkB* strains showed no change in reduced iron, while the Δ*eetA*/*B* and Δ*ndh2* strains had significantly less reduced iron than the WT. (D) Δ*ndh1* mutation was not required for flavin-dependent iron reduction. *n* = 3 for all experiments. All errors shown are SD. Statistical significance was calculated by *t* test (*, *P* < 0.05; ***, *P* < 0.001).

**FIG 6:**
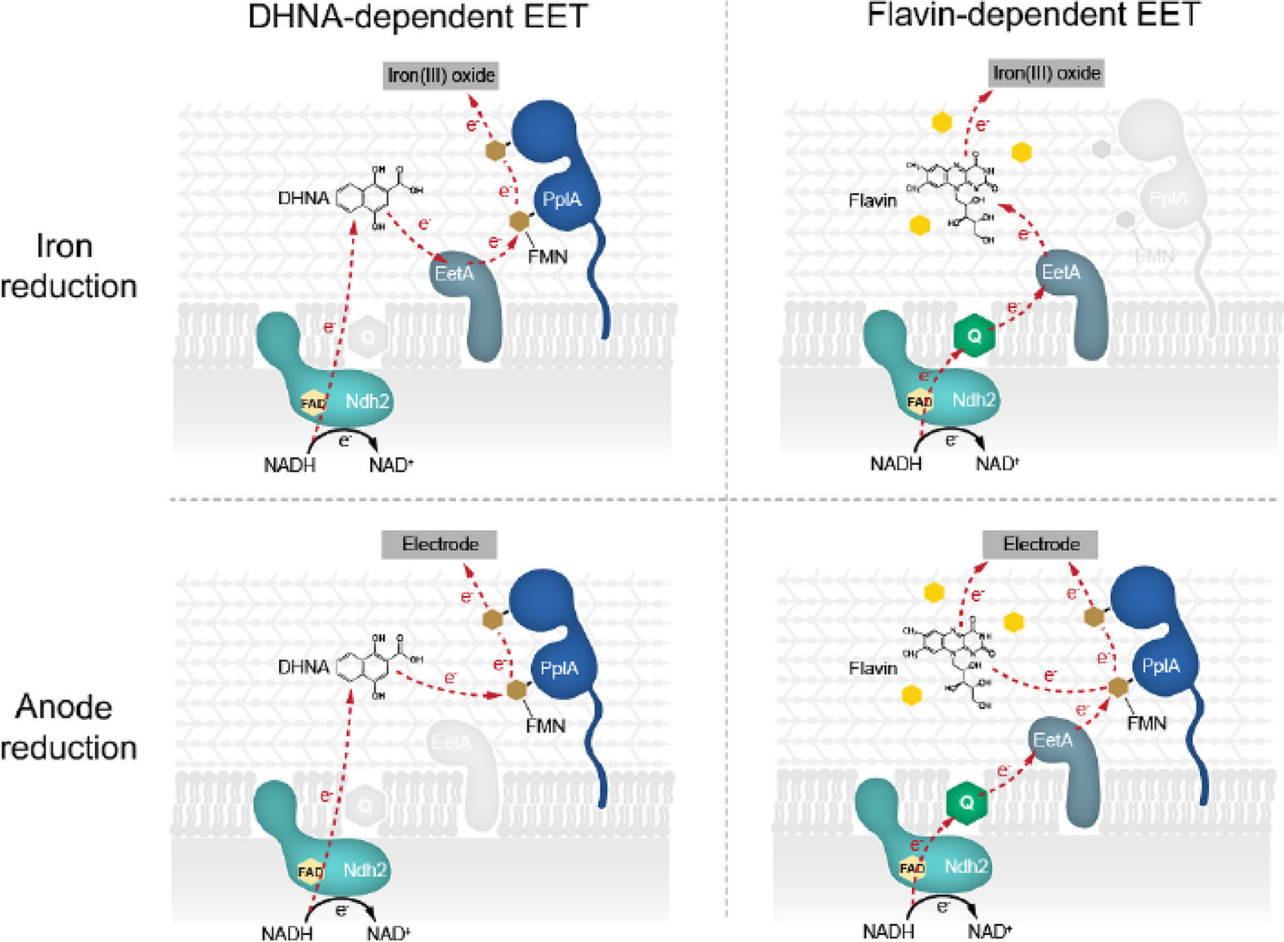
Proposed working model of DHNA- and flavin-dependent EET routes. With the results taken together, this study found that EET can be supported by the presence of exogenous DHNA or flavin species via two distinct routes. Moreover, the two routes appear to be optimized for different time scales and terminal electron acceptors.

Based on our findings that the addition of riboflavin can support robust anode reduction in *L. plantarum*, we next sought to identify which FLEET genes were required to mediate flavin-dependent anode reduction. First, genetic knockouts were tested for their ability to reduce an anode. To maintain consistency with the experiments shown in Fig. 3, we introduced cells to a density of 0.25 OD_600_, which is half the cell density used in the experiments shown in Fig. 1 (OD_600_ = 0.5). In the presence of 2 μg/mL riboflavin, WT cells produced ~11 μA/cm^2^ (Fig. 5A), which is in line with what we expect for half the number of cells used in the prior experiment (−DHNA +RB trend in Fig. 1C). We observed no difference in current production between WT cells and Δ*dmkA* or Δ*dmkB* cells (Fig. 5A). Alternatively, we observed a profound reduction in current production of Δ*pplA*, Δ*ndh2*, and Δ*eetA*/*B* cells compared to the WT (Fig. 5A). The current density seen for these mutants is most similar to the current density observed for heat-killed cells, indicating that biotic EET has been abrogated. Because of potential side reactions with Ndh1, the non-FLEET NADH dehydrogenase, we tested Δ*ndh1* cells for their ability to produce current. Surprisingly, we observed the mutant cells more quickly reach a higher maximal current density than the WT cells (Fig. 5B). This increase in current density observed in Δ*ndh1* cells is likely because Ndh1 maintains redox balance and Δ*ndh1* cells must perform redox balance via Ndh2. Together, we show that flavin-dependent anode reduction likely proceeds through Ndh2 to EetA and then to PplA in a riboflavin-dependent manner. We propose that riboflavin can accept electrons from PplA via the FMN-ylated residues and ultimately to the electrode (Fig. 6).

With the characterization of the role of FLEET genes in flavin-dependent anode reduction, we next sought to address the involvement of these genes in flavin-dependent iron reduction. To assess the effect of genetic knockouts on electron transfer to extracellular iron, we measured iron reduction of FLEET knockout strains in the presence of riboflavin. Interestingly, we found that riboflavin could not support iron reduction in Δ*ndh2* or Δ*eetA*/*B* cells, but riboflavin could support iron reduction in Δ*pplA*, Δ*dmkA*, Δ*dmkB*, and WT cells (Fig. 5C). The lack of effect observed in Δ*pplA* cells is surprising because it has such a strong phenotype under anode reduction conditions. We hypothesize that the PplA is not required for flavin-dependent iron reduction due to the presence of an unknown oxidoreductase or an unknown route of extracellular electron transfer to ferric oxides. Consistent with reactor conditions, Δ*ndh1* cells were found to have no significant change in iron reduction compared to WT cells in the presence of riboflavin (Fig. 5D). To address the potential of nonspecific effect of gene knockouts, we complemented mutant strains where possible. We found that in a Δ*dmkA* Δ*ndh1* Δ*ndh2* strain, complementation of Ndh2 resulted in iron reduction comparable to WT cells (Fig. S6C). Additionally, we found that in a Δ*eetA*/*B* strain, neither EetA nor EetB complementation could restore WT levels of riboflavin-dependent iron reduction (Fig. S6D). The unsuccessful rescue of the Δ*eetA*/*B* mutant is likely due to a combined function of EetA and EetB, rendering a single complement insufficient. Considering the data presented, we propose a novel route of flavin-based electron transfer that initiates from the oxidation of NADH by Ndh2 and proceeds through EetA to extracellular flavin to reduce insoluble iron oxide. Alternatively, in the case of an anode, electrons are transferred to PplA and ultimately to the anode (Fig. 6 and Table 2).

## DISCUSSION

This study investigated the relative importance and mechanism of riboflavin and quinones for EET in *L. plantarum*. We found that both DHNA and riboflavin can independently support EET under minimal medium conditions but that riboflavin requires pregrowth with a quinone. Mechanistically, we observed that DHNA functions as a robust extracellular electron shuttle that likely receives its electrons from Ndh2 or PplA. Moreover, we find that Ndh2, PplA, and EetA are required for anode reduction via riboflavin, but PplA is not required to reduce iron. Although electron transfer from free FMN to DHNA is thermodynamically unfavorable, bound protein-incorporated FMN has been shown to exhibit a drastically different redox potential (27). The electrochemical properties of the FMN residues on PplA have yet to be elucidated. Conversely, we found that riboflavin can also function as an electron shuttle—albeit less efficiently—and that it is likely being converted to FMN by cells to support EET to an anode. Together, this study highlights novel routes of electron transfer that depend on both the redox mediator and the terminal electron acceptor (redox potential and accessibility) (Fig. 6).

This work illuminates several aspects of flavin-dependent EET, but leaves some questions open. While one goal for this study was to minimize differences in cellular status before assaying iron and anode reduction, we still are left with a difference between flavin-dependent iron and anode reduction. This difference is relatively surprising as riboflavin facilitates reduction of iron and an electrode is kinetically favorable, as evidenced by a notable difference in midpoint potentials (28). We hypothesize this difference could be due to two variables: the nature of the terminal electron acceptor or the bioavailability of riboflavin. One such side reaction could be the result of the reactor setup we use for these experiments as it is maintained at a microaerobic state by N_2_ bubbling, but riboflavin has been shown to donate electrons to oxygen at the oxic-anoxic interface in other organisms (29). Finally, it is entirely possible that PplA is not required for flavin-dependent iron reduction due to the functioning of an uncharacterized extracellular flavinated reductase powered by EET.

As a nomadic species that inhabits plant leaves, food/feed, soil and the gastrointestinal tract of various mammals, *L. plantarum* must adapt to diverse environmental niches (30). The identification of EET activity in LABs by this study and others poses the following question: why would an iron-tolerant and great fermenter maintain an EET pathway? One explanation is that *L. plantarum* may utilize multiple routes as a means of increasing the likelihood of the microbe to perform EET and thus enhance fitness (9). Additionally, *L. plantarum*, among other *Lactobacillus* species, exhibits niche-specific genetic alterations that have been linked to this organism's ability to alter its metabolism and regulate the composition of the surrounding microbial community (31–33). This study characterized the ability of *L. plantarum* to utilize biomolecules commonly released by microbes to support EET via overlapping, but unique mechanisms. We assert that multiple EET routes have been maintained by *L. plantarum* to support survival in diverse environments, and which route is preferred is dependent on the availability of redox active molecules in the local environment and the cellular status (i.e., prior exposure of flavin-dependent EET to a quinone to increase the electron flux). Moreover, this study has begun to characterize the metabolic hierarchy utilized by *L. plantarum* to quickly adapt to local conditions.

Multiple studies have highlighted the probiotic benefits of Lactiplantibacillus plantarum, such as, reducing host epithelial inflammation, altering host lipid metabolism, and exhibiting antihyperglycemic potential (34–36). Additionally, it is increasingly evident that microbes in the gut microbiome share and differentially utilize secreted factors like quinones and flavins to survive (37). We believe that the discovery of EET supported by flavins is of significant importance to understanding the dynamics of interspecies microbial energetics within the gut microbial community. EET activity can have a significant impact on microbial gut colonization as evidenced by decreased CFU of EET mutants in studies of L. monocytogenes and E. faecalis (6, 38). Moreover, since EET can facilitate increased fitness and colonization, this suggests that EET-active probiotics could have increased effectiveness. It appears that utilizing both DHNA- and flavin-dependent EET routes could enable *L. plantarum* to persist and thrive in complex environments and gain an energetic advantage over nonelectrogenic microbes.

## MATERIALS AND METHODS

### Microbial cultivation.

Lactiplantibacillus plantarum NCIMB8826 was obtained from Maria Marco. Bacterial strains were grown in MRS (HiMedia Laboratories) from glycerol stocks overnight at 37°C. Fresh overnight cultures were inoculated in mMRS (mannitol as carbon source) at an OD_600_ of ~0.1 and grown for 16 to 18 h at 37°C. The composition of mMRS is as follows unless otherwise noted: 20 mg/mL d-mannitol (Sigma-Aldrich), 1% Tween 80 (Sigma-Aldrich), 10 mg/mL protease peptone (Gibco), 5 mg/mL yeast extract (Sigma-Aldrich), 11.48 mM dibasic potassium phosphate (Avantor Performance Materials), 61 mM sodium acetate trihydrate (Sigma-Aldrich), 8.83 mM tribasic ammonium citrate (Alfa Aesar), 0.83 mM anhydrous magnesium sulfate (Sigma-Aldrich), 0.3 mM manganese sulfate monohydrate (Sigma-Aldrich), 2 mM ferric ammonium citrate (Sigma-Aldrich), and 20 μg/mL 1,4-dihydroxy-2-naphthoic acid (DHNA) (Sigma-Aldrich). Culture medium was supplemented with 10 μg/mL erythromycin (Sigma-Aldrich) or 10 μg/mL chloramphenicol (Sigma-Aldrich) when specified.

### Creation of gene deletions and complementation in *L. plantarum* NCIMB8826.

The strains, plasmids, primers and DNA fragments used in this study are listed in Table S1 in the supplemental material.

The *L. plantarum* NCIMB8826 wild type and *pplA* and *ndh2* deletion mutants were kindly provided to us by Maria Marco (9). *L. plantarum* NCIMB8826 *dmkA*, *dmkB*, *ndh1* deletion mutants were constructed by using the CRISPR-Cas9 toolbox according to Huang et al. (26) Briefly, the upstream and downstream homologous arms were amplified from genomic DNA of *L. plantarum* (see Table S1 for primers), along with single guide RNA (sgRNA) fragments. Both fragments were cloned in ApaI-XbaI-digested pHSP02 editing plasmid to create pSL08 (for *dmkA* knockout), pSTS04 (for *dmkB* knockout), and pSL47 (for *ndh1* knockout) by Gibson assembly (39). For CRISPR editing, *L. plantarum* strain harboring helper plasmid pLH01 was induced with 100 ng/mL sakacin P peptide (GenScript, Piscataway, NJ) to express RecE/T and made electrocompetent. The editing plasmids were then delivered into *L. plantarum* NCIMB8826 by electroporation. The transformed cells were spread on MRS plates containing 10 μg/mL erythromycin and 10 μg/mL chloramphenicol to screen for the deletion mutants. Colony PCR was performed on single colonies to confirm the deletion of the target gene (see Table S1 for primers). Following deletion confirmation, the plasmid was removed via serial growth in nonselective medium.

Gene complementation was achieved based on the pSIP403 backbone (40). The strains harboring pSL39 (*eetA* complement), pSL40 (*eetB* complement), and pSL93 (*ndh2* complement) were induced with 50 ng/mL sakacin P (GenScript, Piscataway, NJ) at 37°C overnight.

### Assaying reduction of iron(III) oxide nanoparticles.

To monitor their ability to reduce iron(III), 3-mL cultures of *L. plantarum* grown in mMRS were grown overnight. The cells from these cultures were pelleted by centrifugation at 4,000 × *g* for 10 min at 4°C and washed with phosphate-buffered saline (PBS) two times. The washed cells were resuspended in PBS to an OD_600_ of 2.0. These cell suspensions were combined with an equal volume of assay master mix to yield samples with a final volume of 0.5 to 1 mL. The assay master mix contained 40 mg/mL d-mannitol, 40 μg/mL DHNA, 4 mM ferric oxide (<50-nm nanoparticle) (Sigma-Aldrich), and 2× PBS (pH 7.4). The samples were introduced into an anaerobic chamber maintained between 30°C to 34°C and were incubated for 24 h before iron reduction was measured. To prevent oxidation of iron upon oxygen exposure, samples were diluted 1:1 in 0.5 M HCl while in the anaerobic chamber. After removal from the anaerobic chamber, the samples were centrifuged at 4,000 × *g* for 10 min at 4°C, and the cell-free supernatant was collected. Fifty microliters of supernatant was added to 200 μL of 2 mM ferrozine in HEPES buffered to pH 7. After 5 min of incubation in the dark, the absorbance was measured at 562 nm (*A*_562_). The iron concentration was calculated by comparison of the measured *A*_562_ to a standard curve of FeSO_4_ ranging from 0.4 to 0.025 mM.

### Bioelectrochemical measurements.

All bioelectrochemical measurements were performed on VSP-300 potentiostat (Biologic). Unless otherwise noted, all measurements were performed using a carbon felt (Alfa Aesar) platinum electrode (Alfa Aesar) poised at 0.2 V versus Ag/AgCl in 3 M KCl for chronoamperometry. Current measurements were collected every 36 s for the duration of the experiment. Cyclic voltammetry was performed from −0.7 to 0.7 V at a scan rate of 5 mV/s. We used a two-chamber 3-electrode setup separated by a cationic membrane. The working chamber contained filter sterilized PBS (pH 7.4) and 20 mg/mL mannitol. The medium was supplemented with 20 μg/mL DHNA, 2 μg/mL riboflavin, or both when specified.

### Medium swap experiment.

Cells were grown and washed as noted previously before being injected into bioelectrochemical reactors containing PBS and 20 mg/mL mannitol. A graphite rod electrode was poised at 0.2 V versus Ag/AgCl, and chronoamperometric measurements were collected every 36 s. After ~3 h, current production leveled out and 2 μg/mL riboflavin or 20 μg/mL DHNA was added. After the peak current was achieved and maintained (~14 h), cells were collected, washed, and kept on ice until reactors with fresh medium without riboflavin were purged. Cells were injected into the new reactors, and chronoamperometry was performed. After ~18 h, 2 μg/mL riboflavin or 20 μg/mL DHNA was added back to the reactors.

### Data availability.

All the data generated in this study, plasmids, and strains can be provided upon request submitted to the corresponding author (Caroline M. Ajo-Franklin, cajo-franklin@rice.edu) for at least 5 years following the publication date. The transfer of the material will be initiated within two weeks from the first request.
