# Supplementary material for: The Differing Roles of Flavins and Quinones in Extracellular Electron Transfer in Lactiplantibacillus plantarum
Source: Appl Environ Microbiol. 2022 Dec 19;89(1):e01313-22. doi: 10.1128/aem.01313-22 (PMC9888254; doi:10.1128/aem.01313-22)
Supplement: Supplemental file 1 — Supplemental material. Download aem.01313-22-s0001.pdf, PDF file, 0.8 MB [file aem.01313-22-s0001.pdf]

Supplemental Information
Figures

Supplementary Table 1: Primers and DNA constructs used in this study. Listed in the table are the DNA constructs used in the study. Included are primer names, sequences, and description of their purpose**.** *Gray color shows the overhang of each primer for assembly

Construct	Primer Name	Sequence (5'-3')	Description
pSL39	*eetA*-F	tatataggagtatgattcccatgcgtaaattacggacaatgatcc	Amplify *eetA* from L. *plantarum*'s genome DNA
	eetA-R	ccttcgaacccggggtaccgaattcctcgagtctagactactccgtaactaaaccattaccg	Amplify *eetA* from L. *plantarum*'s genome DNA
pSL40	*eetB*-F	tatataggagtatgattcccatgaataattcatttgaacgatcaaacaaaact	Amplify *eetB* from L. *plantarum*'s genome DNA
	eetB-F	ccttcgaacatatggtctcaaattcctcgagtctagattactcctttgtttgatctggatggt	Amplify *eetB* from L. *plantarum*'s genome DNA
pSL93	*ndh2-F*	atatataggagtatgattcccatggcaaagaaaaatattgtcgttgtcg	Amplify *ndh2* from L. *plantarum*'s genome DNA
	ndh2-R	ccttcgaacccggggtaccgaattcctagcgggttttaacaccgtcg	Amplify *ndh2* from L. *plantarum*'s genome DNA
pSL08	Δ*dmkA*_up_arm_F	aaagtggcaccgagtcggtgcttttttgagtgttggacttatcgcgtgct	Upstream homologous arm for *dmkA* deletion
	Δ*dmkA*_up_arm_R	gtttctttcacctgcaccgccacactagctgccttggcatt	Upstream homologous arm for *dmkA* deletion
	Δ*dmkA*_down_arm_R	gcggtgcaggtgaaagaaac	Downstream homologous arm for *dmkA* deletion
	Δ*dmkA*_down_arm_R	cacatctttttctaaactagggcccgcaactgcaaaccgccttaa	Downstream homologous arm for *dmkA* deletion
	Δ*dmkA*_sgRNA	tggacatactatgatatattctaga**cttaaagcccgccagtacgg**gttttagagctagaaatagcaagttaaa ataaggctagtccgttatcaacttgaaaaagtggcaccgagtcggtg	DNA fragment containing sgRNA sequence targeting *dmkA* (**bold text** indicates 20nt crRNA)
	Δ*dmkA*_check_F	gggcgaagttgttgacgaac	Check *dmkA* deletion
	Δ*dmkA*_check_R	gaagcaccgactcatgacca	Check *dmkA* deletion
pSL47	Δ*ndh1*_up_arm_F	aaagtggcaccgagtcggtgcttttttgagtgtccgcgttggtttttgtc	Upstream homologous arm for *ndh1* deletion
	Δ*ndh1*_up_arm_R	aagaccgttcctaaatgcgcaacaagaatgatttcatcctcagttgg	Upstream homologous arm for *ndh1* deletion
	Δ*ndh1*_down_arm_F	aggatgaaatcattcttgttgcgcatttaggaacggtcttc	Downstream homologous arm for *ndh1* deletion
	Δ*ndh1*_down_arm_R	cacatctttttctaaactagggcccctctattggctgcgcgaaac	Downstream homologous arm for *ndh1* deletion
	Δ*ndh1*_sgRNA	tggacatactatgatatattctaga**tgctgtggtttacagtgacg**gttttagagctagaaatagcaagttaaaat aaggctagtccgttatcaacttgaaaaagtggcaccgagtcggtg	DNA fragment containing sgRNA sequence targeting *ndh1* (**bold text** indicates 20nt crRNA)
	Δ*ndh1*_check-F	gcgtggcgttttagctgaat	Check *ndh1* deletion
	Δ*ndh1*_check-R	atttctggggaacgcttggt	Check *ndh1* deletion
pSL51	Δ*ndh2*_up_arm_F	aaagtggcaccgagtcggtgcttttttgagtccttgaccgtgattgcgaa	Upstream homologous arm for *ndh2* deletion
	Δ*ndh2*_up_arm_R	gtaaacaccagcaaaccccg	Upstream homologous arm for *ndh2* deletion
	Δ*ndh2*_down_arm_F	cggggtttgctggtgtttaccttggacactggtggtacgg	Downstream homologous arm for *ndh2* deletion
	Δ*ndh2*_down_arm_R	cacatctttttctaaactagggcccttatgcatgacgccaccagt	Downstream homologous arm for *ndh2* deletion
	Δ*ndh2*_sgRNA	tggacatactatgatatattctaga**ggcattgaaactaacccccg**gttttagagctagaaatagcaagttaaa ataaggctagtccgttatcaacttgaaaaagtggcaccgagtcggtg	DNA fragment containing sgRNA sequence targeting *ndh2* (**bold text** indicates 20nt crRNA)
	Δ*ndh2*_check-F	cgatgccactattttcgcgg	Check *ndh2* deletion
	Δ*ndh2*_check-R	agatcgtgacgtgggtgaac	Check *ndh2* deletion

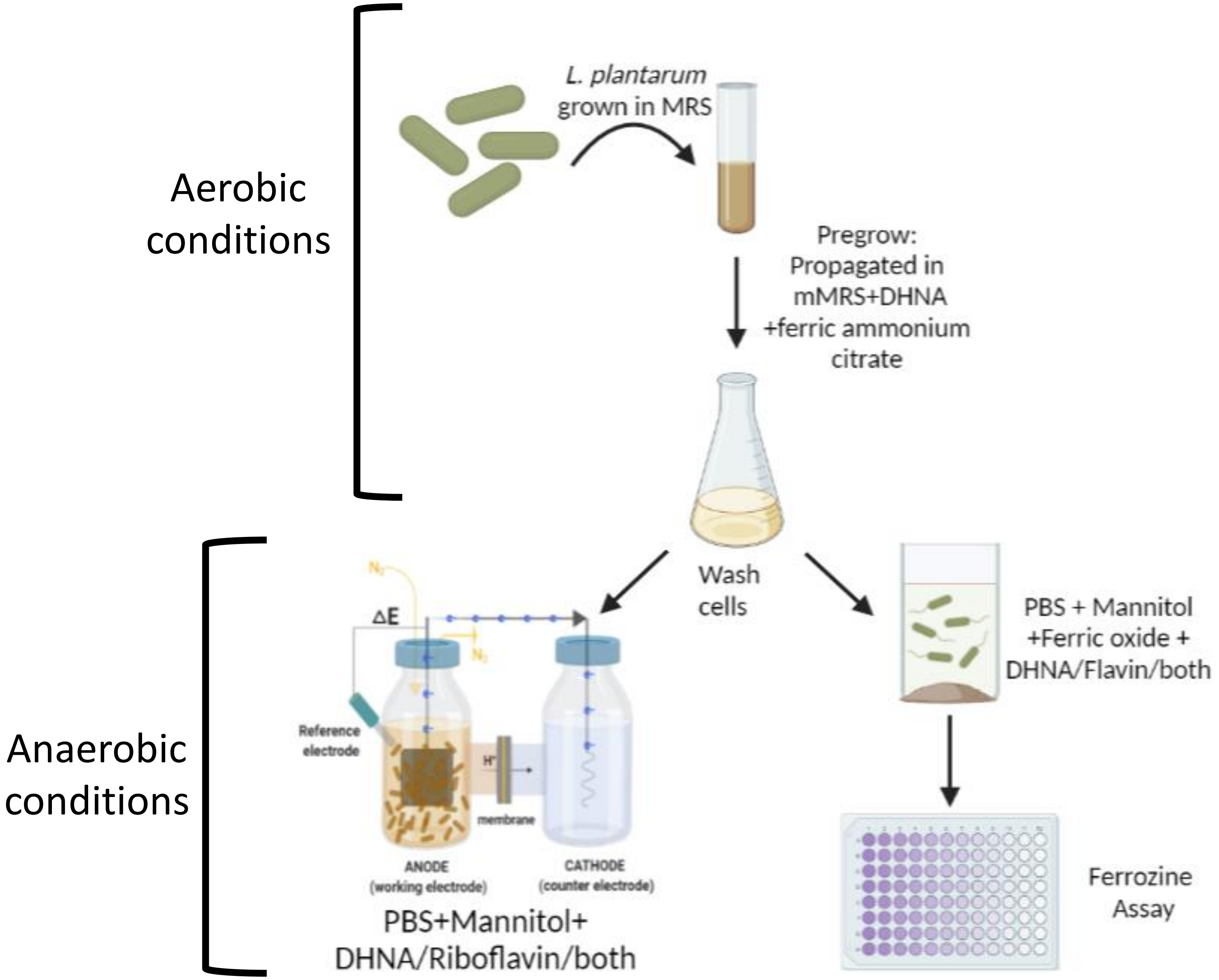

SI Figure 1: Experimental approach and growth conditions. L. plantarum was grown aerobically from a frozen stock in MRS media. Next, mMRS supplemented with DHNA and/or ferric ammonium citrate was inoculated with cells from MRS culture. Cells were washed with PBS before use in anaerobic iron reduction or anode reduction experiments. This figure was made using biorender.com.

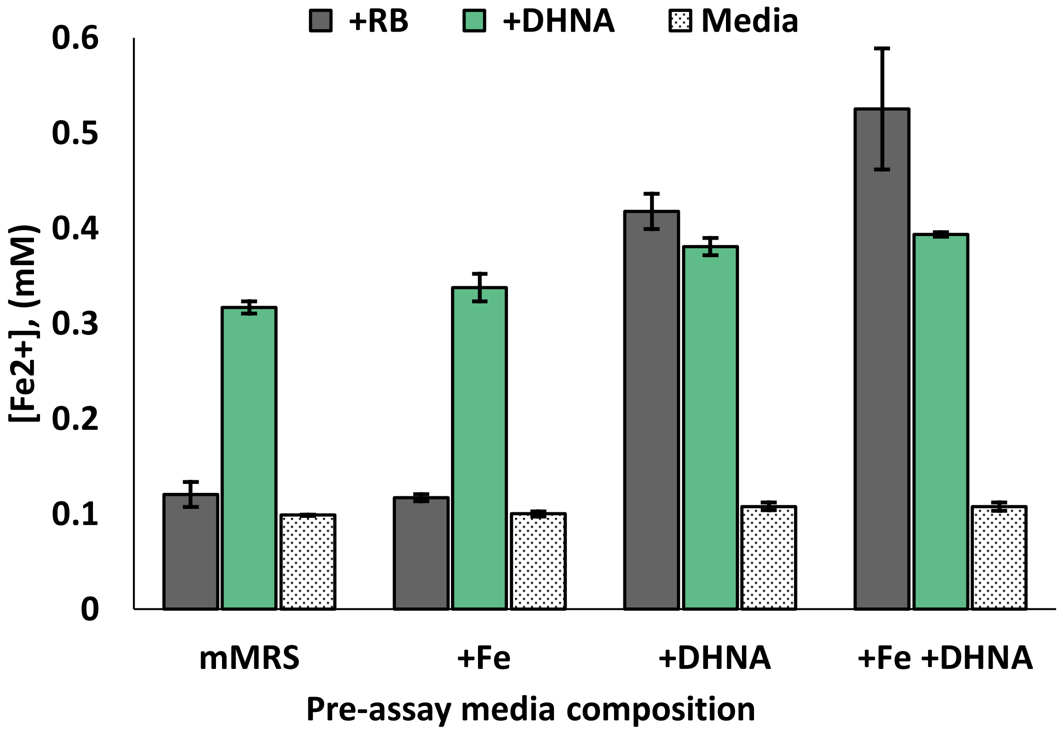

SI Figure 2: Pre-assay media supplementation affects Flavin-dependent iron reduction. Concentration of Fe^{2+} produced from ferric oxide nanoparticles after 24 hours anaerobic incubation in PBS + 20 µg/mL Mannitol (pattern) alone or supplemented with DHNA (green), Riboflavin (grey). Pre-assay media composition indicates overnight culture source contained exogenous DHNA, ferric ammonium citrate, or both. Error is shown as standard seviation.

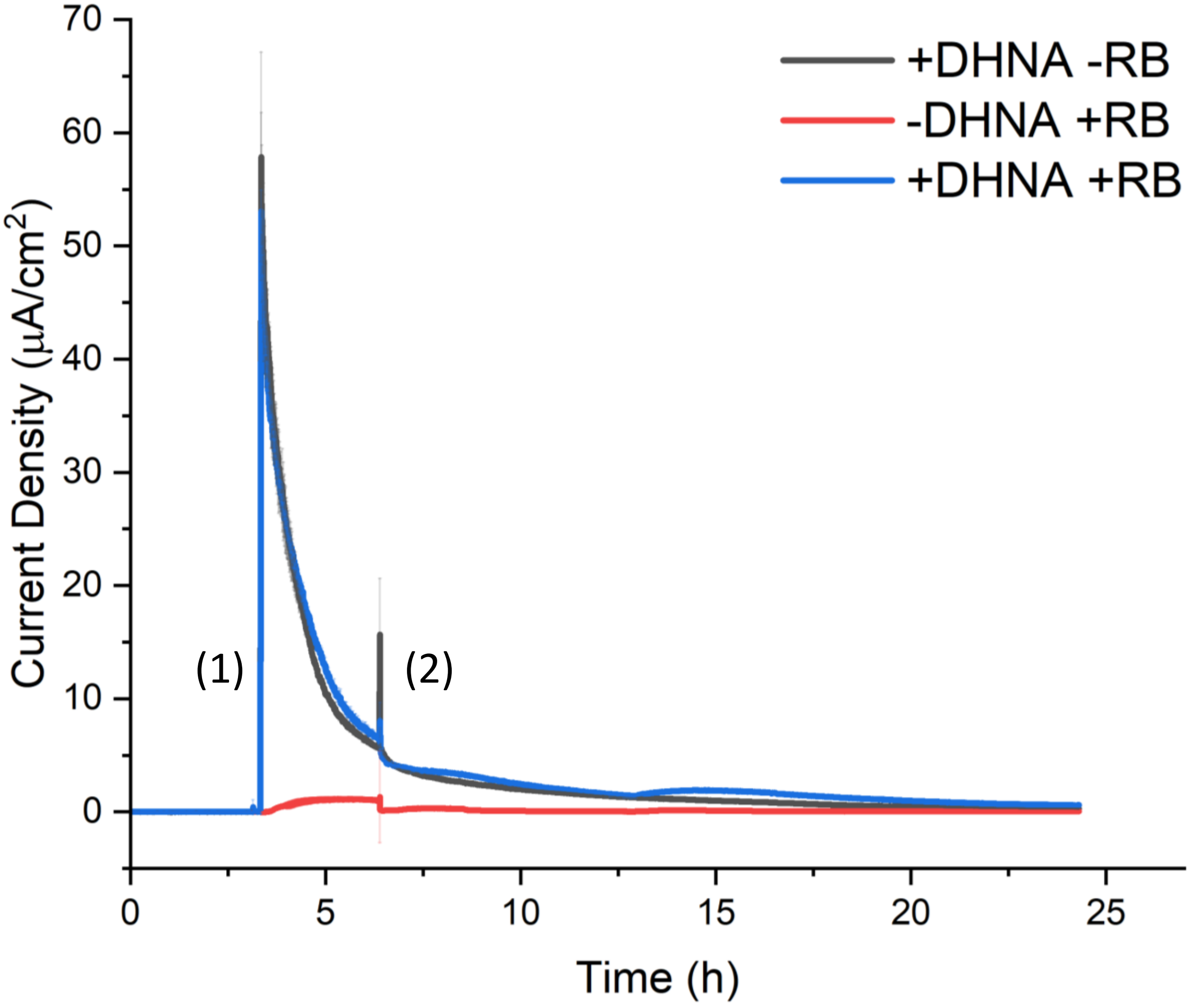

SI Figure 3: Heat-killed cells cannot produce current.
Chronoamperometry measurements were taken every 36s for 24h. Abiotic media (PBS + Mannitol) shows minimal background. After 3h, supplemental compounds were added to reactors (1). The spike observed in +DHNA-RB and +DHNA+RB is the result of abiotic oxidation of DHNA. Once most of the DHNA was abiotically oxidized, heat-killed WT *L. plantarum* was added to an OD600 of 0.5 (2). Error is shown in standard deviation. N=2 reactors.

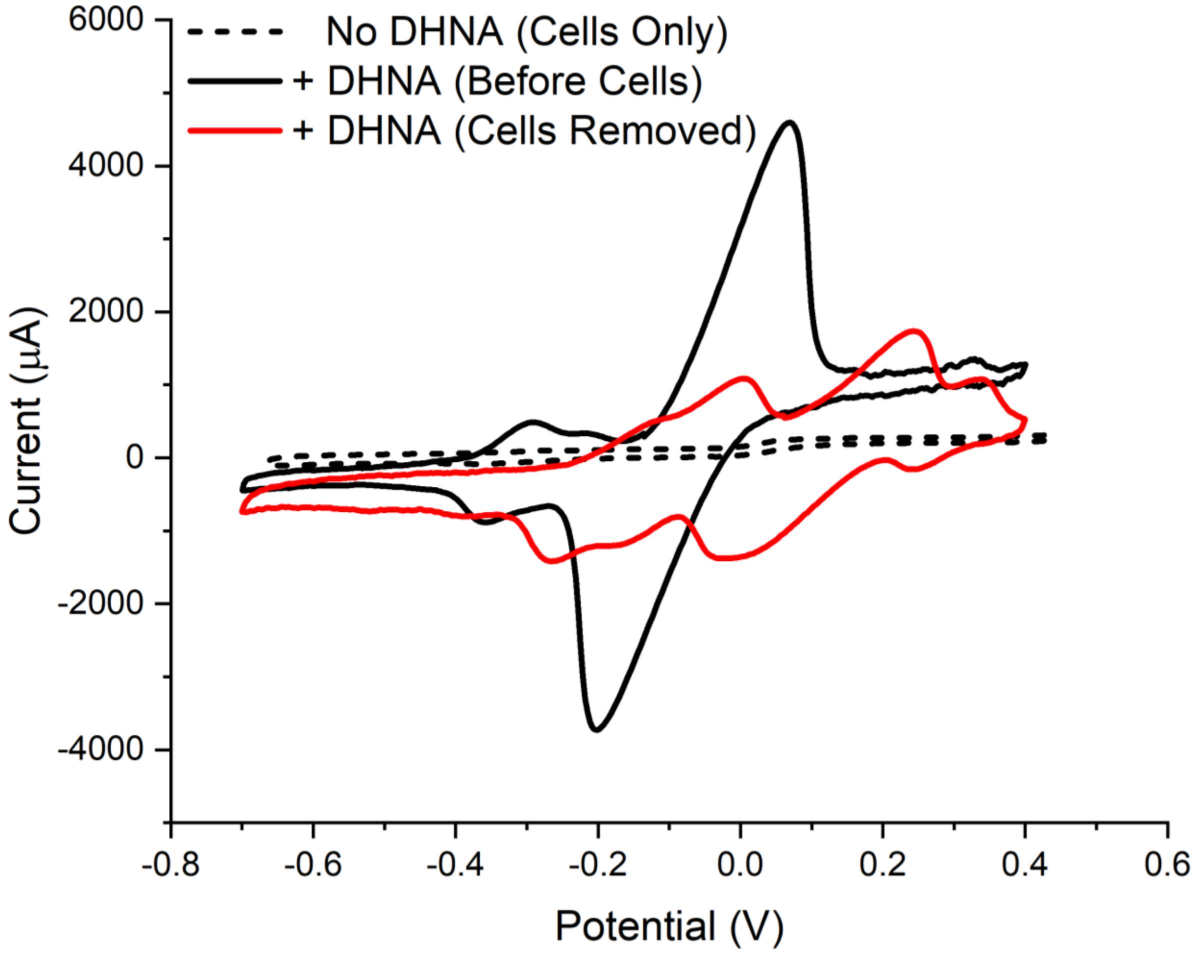

SI Figure 4: Cell-free spent DHNA containing media maintains similar electrochemical profile to cell containing media. Cyclic voltammetry was performed prior to the addition of cells (black line). *L. plantarum* cells were added and allowed to metabolize an electrode poised at 0.2V vs Ag/AgCl (3M KCl) for 24 h. After 24 h, media was filter sterilized, purged for 3 h and an additional cyclic voltammetry measurement was taken. In the presence of DHNA, 3 reduction peaks are observed between -0.1V and +0.3V (red line). As a control, cells grown in the absence of DHNA lack all peaks observed in conditions containing DHNA.

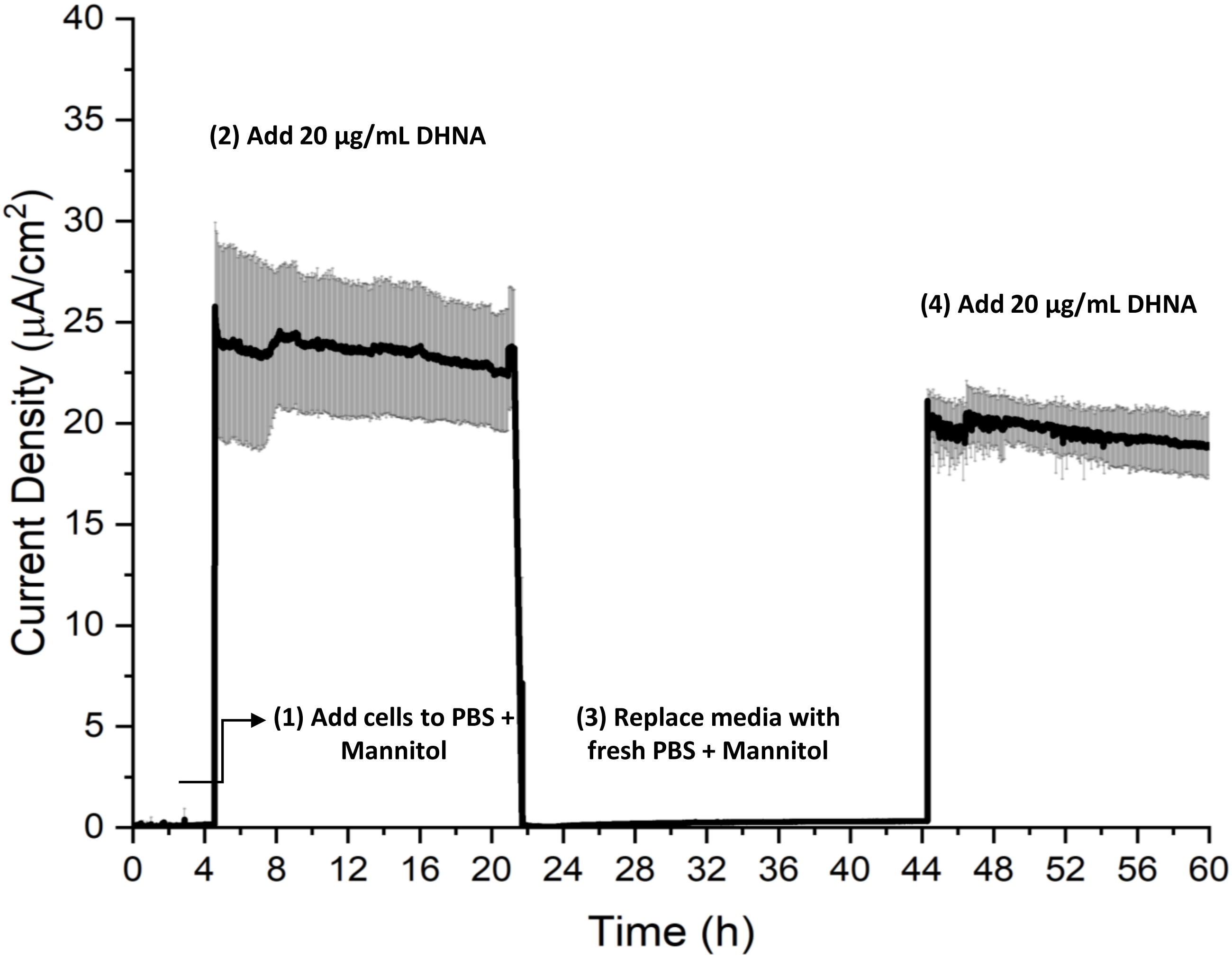

SI Figure 5: Media swap confirms DHNA acts as an electron shuttle. (1) Cells were inoculated in bioelectrochemical reactors containing graphite rod electrode poised at 0.2V vs Ag/AgCl. (2) After 4h, DHNA was added to a concentration of 20 µg/mL. (3) cells were collected and washed 2x with PBS before being returned to reactors containing fresh media lacking DHNA. (4) After ~24h, DHNA was again added to a concentration of 20 µg/mL. Error shown is standard deviation and N=3 bioreactors.

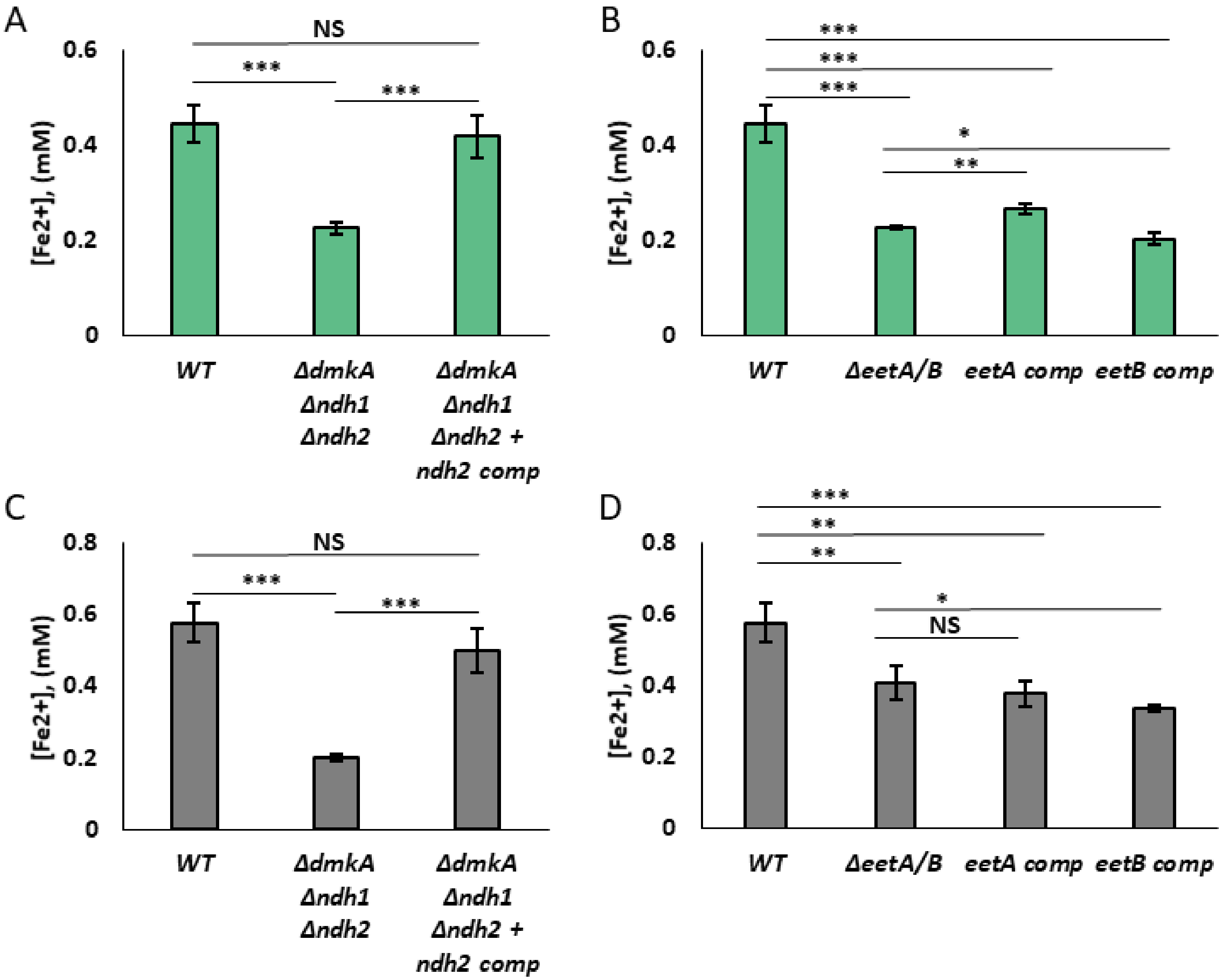

SI Figure 6: Ndh2 complementation restores DHNA- and riboflavin-dependent EET. Concentration of Fe^{2+} produced from ferric oxide nanoparticles after 24 hours anaerobic incubation in PBS + 20 µg/mL mannitol supplemented with DHNA (A&B) or Riboflavin (C&D). Cells were pregrown with DHNA and ferric ammonium citrate. Complementation of EetA, EetB, and Ndh2 was accomplished by induction with 50 ng/mL Sakacin P in the overnight culture. N= 3 biological replicates. Error is shown as standard deviation.

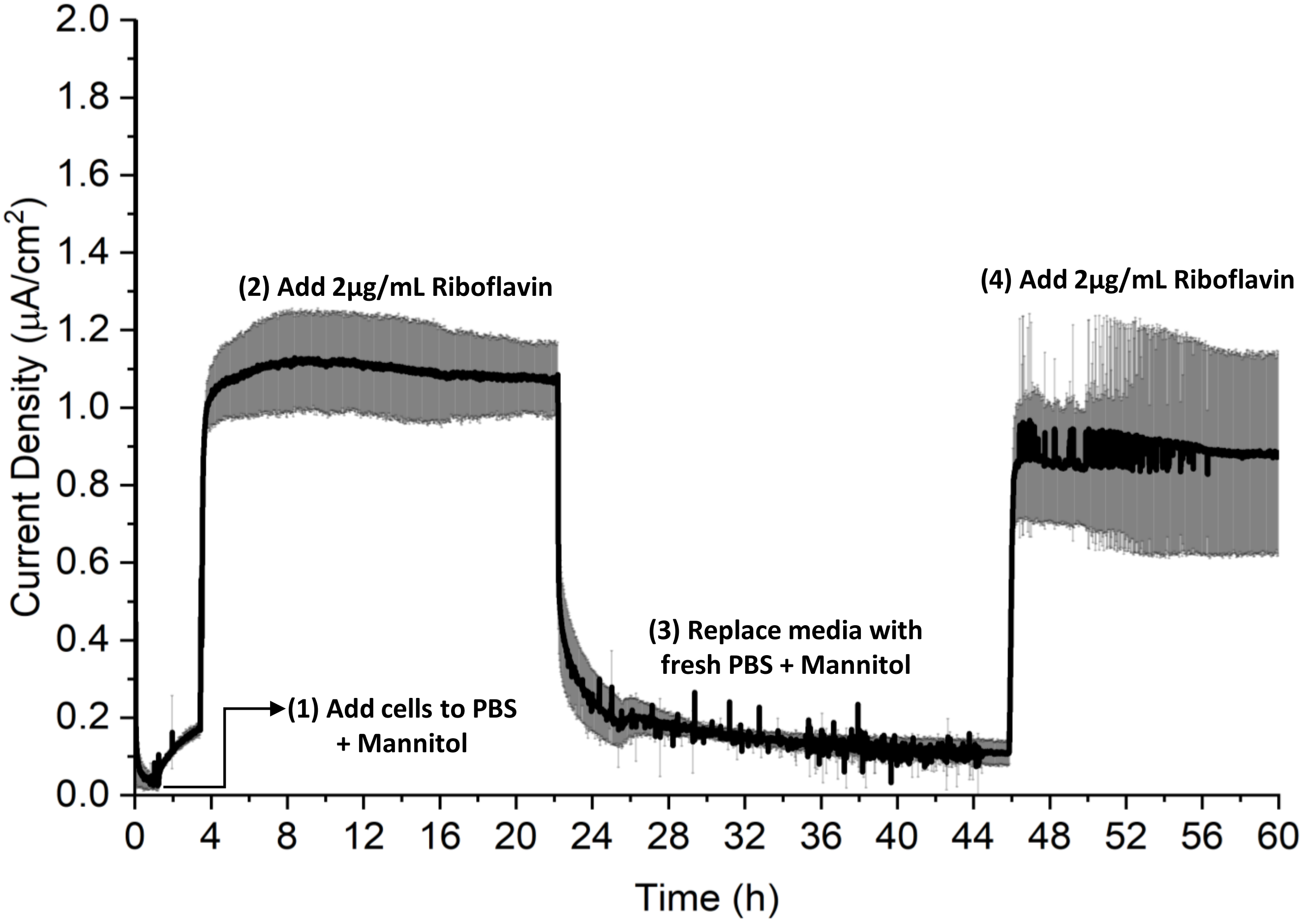

SI Figure 7: Media swap confirms riboflavin can act as a poor electron shuttle. (1) Cells were inoculated in bioelectrochemical reactors containing graphite rod electrode poised at 0.2V vs Ag/AgCl. (2) After 4h, Riboflavin was added to a concentration of 2 μg/mL. (3) cells were collected and washed 2x with PBS before being returned to reactors containing fresh media lacking Riboflavin. (4) After ~24h, Riboflavin was again added to a concentration of 2 μg/mL. Error shown is standard deviation and N=3 bioreactors.
